# Exploring young people’s interpretations of female genital mutilation in the UK using a community-based participatory research approach

**DOI:** 10.1186/s12889-020-09183-6

**Published:** 2020-07-20

**Authors:** Saadye Ali, Nick de Viggiani, Aida Abzhaparova, Debra Salmon, Selena Gray

**Affiliations:** 1 Faculty of Health and Social Sciences, Aylesbury Campus, Stoke Mandeville Hospital, Aylesbury, HP21 8AL UK; 2 Centre for Public Health and Wellbeing, The University of the West of England (UWE, Bristol), Frenchay Campus, Coldharbour Lane, Bristol, BS16 1QY United Kingdom; 3 Department of Geography and Environmental Management, The University of the West of England (UWE, Bristol), Frenchay Campus, Coldharbour Lane, Bristol, BS16 1QY United Kingdom; 4 Schools of Health and Sciences, City University London, London, EC1V 0HB UK

**Keywords:** Female genital mutilation (FGM), Female genital cutting (FGC), Second-generation, Beliefs, Attitudes, Migration, UK, Body image, Identity, Community-based participatory research (CBPR)

## Abstract

**Background:**

Female genital mutilation (FGM) is a deeply-rooted cultural practice mainly undertaken in Africa, the Middle East and Asian countries. Evidence to date suggests that although first-generation migrants to the West are abandoning FGM, the custom continues in some places, albeit in small numbers. This study examined how young people living in FGM affected communities in the United Kingdom (UK), interpreted and explained FGM.

**Methods:**

A community-based participatory research (CBPR) approach was used to recruit and train nine young people aged 15–18 as co-researchers. These comprised eight females and one male from second-generation FGM affected communities, living in Bristol. The co-researchers then undertook focus groups and semi-structured interviews with twenty participants aged 13–15 living in Bristol, Cardiff and Milton Keynes. The qualitative data from the training workshops, interviews and focus groups were collected and analysed using thematic analysis.

**Results:**

There were conflicting views among participants. Some perceived FGM as a historical tradition that was of very little, if any, relevance to them. In contrast, others perceived that the more archaic, cultural interpretation of FGM, more commonly shared by older generations, had been supplanted by a new form of FGM, which they believed to be a safe procedure, made so by the availability of highly-trained, qualified doctors and better equipment in the UK. Participants spoke of challenges encountered when attempting to raise the issue of FGM with parents. Nevertheless, they acknowledged that– being born and raised in the UK – enabled them to talk openly and to challenge others.

**Conclusion:**

Future strategies to address and prevent FGM in the UK will require a public health approach that is holistic, intersectional and empowering. Such measures should be relevant to young people born and raised in the UK who interpret FGM differently to previous first-generation migrant relatives and communities. Tackling FGM requires a shift away from a principal preoccupation with harm reduction and criminalisation towards collaboration and active dialogue with communities, in positive and productive ways that acknowledge and engage issues of identity, race, gender, and generation, enabling people affected by FGM to take control of their health and well-being.

## Background

Female genital mutilation (FGM) is an overarching term used to define cultural practices that result in the modification of female genitalia for non-medical reasons. The World Health Organisation (WHO) distinguishes four types of FGM [1] according to the severity of the procedure. Type I, also known as clitoridectomy, is the partial or total removal of the clitoris and/or the prepuce; Type II, or excision, is the partial or total removal of the clitoris and the labia minora, with or without excision of the labia majora; Type III, also known as infibulation, is the narrowing of the vaginal orifice with the creation of a covering seal by cutting and repositioning of the labia minora and/or the labia majora, with or without excision of the clitoris, leaving a small hole to allow voiding of the bladder and menstruation. Type IV includes all other practices in the genital area, with varying degrees of severity and without medical reasons. FGM is associated with several health implications, both long term and short term [2, 3], including physical, psychological, sexual and reproductive complications.

According to a 2016 statistical report by UNICEF [4], the global incidence of FGM has declined over the last three decades. However, UNICEF argues that, due to population growth, the total number of girls and women affected by FGM globally will rise significantly in the next 15 years, if effective action is not taken to prevent and stop it. Immigration has made the issue topical in Western countries, as FGM affected communities settle in the West. Although the exact number of women and girls living in Europe who have undergone the procedure is not known [5], it is estimated that there are around 103,000 such women aged 15–25 and 10,000 aged 0–14 living in England and Wales today [6, 7].

FGM is often performed on young girls between 4 and 12 years, at the later ages to signify their transition to womanhood [8]. It is important to note that the age when FGM is performed varies, ranging from a few days old to adolescence, adulthood, before marriage and post-partum [9, 10]. There is further diversity in terms of country, tribe, circumstances, and community. Girls living in the United Kingdom are most likely to have been “cut” between the ages of 5 and 10 [11, 12].

Public opposition to FGM has generated a rise in anti-FGM activists’ campaigns and prohibitive legislation in the UK. The recent case of a woman in London imprisoned for 11 years for subjecting her daughter to FGM suggests the strength of legislative efforts to curtail its practice through penal punishment of those who continue the tradition [13]. Paradoxically, however, although intended to eradicate the custom, these efforts may instead lead perpetrators to develop new strategies to circumvent the law – genital cutting may be undertaken either within hospital settings in their parents’ countries of origin or secretly ‘on the black market’ in the UK [12]. However, there is currently no evidence to suggest that FGM is being carried out in the UK by second-generation immigrants, although this may be due to a lack of research in this field, which would benefit future research study.

### FGM and immigrant cultural identity

According to the International Migration Report, in 2017 an estimated 258 million people around the world were residing in a different country than the one they had been born in, an increase of 49% since 2000 [14]. The concepts of acculturation and assimilation are used to conceptualise the process whereby migrants settle into and become members of their new society [15]. Acculturation is said to be the first stage of assimilation, where two different ethno-cultural groups come into contact for an extended period, leading to cultural changes within both groups [16]. This definition implies that the dominant, as well as the non-dominant cultural groups, are influenced by their intercultural contacts and are eventually transformed to acquire features of each other’s cultural values.

Assimilation is a process whereby immigrants relinquish certain parts of their identity and adapt to the culture of the majority society [17]. There are four significant sub-processes and three ideologies of assimilation, and the ‘popular view’ assumes that immigrants will become assimilated within three generations [18]. This presumes that the first generation often faces difficulties in assimilating – particularly due to the language barrier. Integration experiences of second-generation youth differ from those of the first, simply because the initial settlement barriers, such as language, have been removed [15, 18], and they have been exposed to local forms of education and social relationships from birth [19–21]. The third generation, the first generation’s grandchildren, tend to be completely assimilated [18]. However, this theory can be problematic for some groups, since parents of second-generation immigrants who still adhere to the traditional practices and values of their country of origin may expect the same adherence to the home culture from their second-generation children [21]. As a result, second-generation children may have very demarcated boundaries in their sources of culture transmission and the ways they respond to the bifurcated cultural demands [19].

Research on FGM to date has focused on understanding the beliefs and attitudes of first-generation migrant adults towards the practice [22–25]. Researchers have concluded that migrants gradually leave behind old cultural customs - such as FGM - and take on those of the host community [23–25]. However, the process whereby individuals acquire the beliefs, values and customs of their new host country does not necessarily mean that they will discard the beliefs, values and customs of their country of origin [26–28]. Furthermore, assimilation may be slow or even occasionally non-existent, due to social barriers such as racism and unequal power structures in the host country [29]. Immigrant groups are often subject to discrimination and alienation from mainstream society, prompting them to cling onto and retain the values, traditions and customs of their birthplaces as a form of defence against ostracism and social oppression [28, 29].

This article explores how second-generation young people living in the United Kingdom (UK) – whether directly or indirectly affected by FGM – interpret and understand FGM.

## Methods

The findings presented in this article arise from a PhD project which took a community-based participatory research (CBPR) approach to examine how approaches aimed at preventing FGM can be improved and developed with second-generation young people in the UK [30].

CBPR is a collective, reflective and systematic approach to research where researchers and communities engage as equal partners in the research design and execution, with the goals of educating, improving or bringing about social change [31–33]. Therefore, CBPR aligns with an ontology of participatory reality and an epistemology of experiential and participative knowing, informed by critical subjectivity and participatory transaction [32, 33].

This view acknowledges that learning is constructed by cultural differences and by the context in which it takes place [34]. As a result, access to people’s realities and experiences may be achieved through direct social interaction. In the case of a researcher, social interaction creates active, reciprocal engagement and develops shared meanings and interpretations, meaning that all research is socially situated within its setting, so the researcher must be able to engage and adapt appropriately to the research environment [35, 36]. The premise is that researchers and participants alike bring their individual unique perspectives and values to the research and operate as independent, and interdependent, social beings [31, 32]. In this sense, there is no singular reality of an experience, but a range of perspectives and interpretations – of FGM – that may manifest.

### Accessing the research setting

The research took place in three communities in Bristol, Cardiff and Milton Keynes, selected because each had relatively large Black, Asian and minority ethnic (BAME) communities affected by FGM [6, 7]. Greater London has the largest estimated proportion of people from FGM affected countries (21.0 per 1000 population), followed by Bristol with an estimated 12–15 people per 1000, and 7 per 1000 for both Milton Keynes and Cardiff [6, 37]. The principal researcher, SA had established longstanding relations with FGM affected communities in Bristol and Milton Keynes over a series of years, as a trainer and community development worker, and this PhD research evolved through her relationships with key stakeholders in these communities. Cardiff was chosen due to its near geographical proximity to Bristol, and the availability of a gatekeeper who was willing to engage with the research.

### Recruitment and sampling

Recruitment of participants’ involved two phases, the first of which entailed recruitment of nine young people aged 15–18 from an FGM affected community in Bristol, to train as co-researchers. These recruits would eventually conduct focus groups and interviews with the second wave of younger participants aged 13–15 recruited from three neighbourhoods in Bristol, Cardiff and Milton Keynes. The co-researchers and younger research participants were all born and raised in the UK to first-generation parents. The decision to engage with these specific age groups was twofold. First, due to the lack of published research on FGM involving young people, this project sought to add to the current scholarship by paying specific attention to second-generation young people’s perspectives of the custom. Second, Relationship and Sex Education (RSE) is compulsory in UK secondary schools from the age of eleven onwards, and schools are advised to educate students on FGM as part of this; students are taught about the physical and emotional damage caused by ‘cutting’, as well as UK law in respect of this custom [38]. It was considered that armed with this knowledge, young people aged 13 and above would be able to share their perspectives on this subject.

After much discussion with research colleagues and with key contacts within Bristol’s BAME community organisations, it was decided to attempt to recruit young people to the research via local schools that served communities known to be FGM affected. The principal researcher approached the head teachers of eight schools via email, during 2016, inviting them to become involved as gatekeepers in recruiting young people to the research. Unfortunately, none of these schools replied, despite repeated approaches. Therefore, a pragmatic decision was made to try to access participants via two community organisations, one in Bristol and the other in London — both of which were known to the principal researcher, with whom she had good relations. The manager of the Bristol organisation agreed to meet to discuss the research and acquired permission from the organisation’s trustees for the research to proceed. Participant information was provided for dissemination to families in contact with the organisation, and contact details for families willing to learn more about the research and who signalled interest in participating were elicited. The principal researcher then organised a series of follow-up meetings with families who had expressed interest in the project, which provided the opportunity to discuss the purpose and aim of the research, the methods and approach, potential ethical issues, timescale and to answer their questions. The manager was also a Somali translator, so was able to support this process. The young people were not present at these preliminary meetings and were only approached after parents had agreed they could become involved. Five co-researchers were recruited via the Bristol organisation, and four joined after hearing about the research from their peers. Eventually, nine co-researchers and twelve participants were recruited in Bristol.

Recruitment of participants in Cardiff followed the same process and was undertaken concurrently with Bristol. The London-based organisation agreed to assist and introduced the principal researcher to an organisation that worked with FGM affected communities in Cardiff. However, accessing participants in Cardiff was considerably more challenging since it proved to be more difficult to establish reliable contacts and build relations with the gatekeeper organisation. The process of building trust took 1 year, from November 2016 to October 2017, during which there were repeated visits to Cardiff and sustained interactions, to earn the gatekeeper’s trust. It was important to demonstrate respect and a willingness to learn. Eventually, nine co-researchers and twelve participants were recruited in Bristol.

The co-researcher team was essentially recruited through convenience sampling via the early meetings with the gatekeeper organisations and participants’ families. A purposive, snowball sampling technique was then used [38, 39] to recruit the second phase research participants, via the co-researchers and then via research participants themselves, through acquaintances, peers and friendship networks, which led to the recruitment of three research participants in Milton Keynes.

### Training the co-researchers

Following successful recruitment of the co-researchers, a 12-week period of training was undertaken in 2016–17 to prepare them for undertaking the research with the younger research participants in the three localities. The training took place at a youth centre in the centre of Bristol, a location identified by the co-researchers and their parents for its acceptability and accessibility. A training programme was developed that employed team-building and interactive learning techniques, including drawing and writing approaches. The training was delivered by the principal researcher and an external facilitator experienced in the use of participatory approaches with young people. The training explored with co-researchers the purpose of the research, the CBPR methodology, qualitative research methods – including interviewing, focus groups and qualitative analysis – sexual and reproductive health, clinical, epidemiological and legal features of FGM, safeguarding policy and practice and intercultural communication. This was a collaborative process that enabled the trainees to become progressively engaged as a group, to increase in confidence and to feel empowered to begin to undertake focus groups and qualitative interviewing. It was important that the researcher and facilitator approached and worked with the co-researchers on an equal basis, valuing their status and contribution and strove to build relationships based on trust and reciprocity. The goal was to maximise their involvement through this process [40–42], to prepare them to be effective, credible and confident researchers. As such, they were encouraged and aided to make decisions about the key stages of the project, especially in terms of conveying information about the project in appropriate and accessible language. The co-researchers spent time clarifying and demystifying some of the language of research and concerning the more “academic” language used to discuss FGM. This was illustrated in a discussion about FGM prevention where, when asked to think of interventions aimed at preventing FGM, Uba, a female co-researcher, replied, “No, wait, I have a question: what *are* interventions?” This triggered a valuable discussion on the importance of using appropriate language to develop the interview questions for use in the interviews and focus groups. They also engaged in an extensive discussion of the value of participatory methods as an approach for using with young research participants.

### Data collection

In March 2017, the co-researchers conducted their first two focus groups in Bristol with the first group of research participants. These were audio-recorded and facilitated by two of the co-researchers. The principal researcher was always present but maintained a passive role throughout. Each focus group lasted approximately 2 h and took place in a community centre close to where participants lived. The first focus group involved seven participants, four males and three females, and was facilitated by two of the female co-researchers. The second focus group involved five males and was facilitated by the one male co-researcher. In line with CBPR principles, the decision to conduct a mixed-gender focus group was made by the co-researchers and participants. A third focus group was organised in Cardiff in December 2017, which involved five female participants; however, due to unforeseen circumstances, the co-researchers had to pull out at short notice, so the principal researcher stepped in to facilitate this focus group, to avoid cancelling it.

The focus groups were organised as informal conversations, beginning with icebreakers and general questions to create a relaxed and convivial atmosphere. The co-researchers posed questions such as, ‘what do other people say about FGM?’ framing these in the third person to make the tone of the questioning as non-threatening as possible (See Additional file A for focus group guide). This had the effect of placing emphasis on participants’ perceptions of how *others* relate to or refer to FGM and related issues to avoid eliciting personal disclosures of belief or experience, especially since this was not the intention of the focus groups. In seeking to facilitate full exploration and discussion of these questions and issues, the co-researchers used a range of techniques, including drawing, role play and writing [42–44]. This technique enabled participants to express themselves individually and anonymously ‘on paper’, through images and words or phrases, which were then used anonymously to foster discussion and debate in the group.

Twenty audio-recorded semi-structured interviews were subsequently undertaken between March 2017 and January 2018 with the focus group participants. This period of 10 months was required to fit in with the co-researchers’ and research participants’ busy schedules. These interviews lasted between 50 min and 1 h and were undertaken by one co-researcher, while the principal researcher was present but remained passive. The questions explored individual participants’ beliefs and perceptions of FGM, their sources of knowledge about FGM and the role of family, school, peers and media in their understanding and interpretation of FGM (See Additional file B for interview guide).

The principal researcher’s involvement as an observer was necessary to comply with ethical approval and for safeguarding reasons.

### Ethics approval

This study adhered to the British Educational Research Association ethical guidelines [45] and was approved by the Research Ethics Committee of the Faculty of Health and Applied Science at the University of the West of England, Bristol, UK in August 2016 (reference: HAS.16.07.176). The considerations taken into account are detailed below.

### Safeguarding

Research in this sensitive area required consideration of safeguarding issues. The term ‘safeguarding’ extends beyond the definition of child protection, to include the notion of prevention [46]. This is the need to avert any harmful acts from occurring to a vulnerable third-party, which may be relevant to the researchers hearing any disclosures of FGM risk.

Although participants were not asked to disclose their personal experiences of FGM, the focus of the project meant that there was potential for some participants in the research to have either experienced FGM or to have been at risk of FGM. Robust safeguarding protocols were adhered to, which required working closely with organisations and agencies that supported and advised young people within BAME and FGM affected communities. The project was assisted by a Children’s Safeguarding Lead based at Bristol City Council who was available as a point of referral should this be necessary. Furthermore, the principal researcher was a registered nurse with a Level 3 Safeguarding qualification, and was present throughout all phases of data collection, to respond to potential safeguarding issues.

### Consent

Considering the age of the participants, and due to the nature of the research subject, it was essential to engage and gain consent from parents in this project. The reasons for this were two-fold. Firstly, following conversations with gatekeepers, it was anticipated that young people were more likely to attend meetings and training if their parents had consented for them to do so. Secondly, mothers brought their children to attend the interviews and focus groups. The successful completion of this study was, therefore, attributed to parental involvement, as well as the trust built with the communities.

Parents were provided with information sheets outlining the research aims and purposes and the roles of co-researchers and participants. They were given a two-week ‘cooling-off’ period in which to decide whether they wanted to grant consent for their children to take part. The principle researcher then met with the young people who had expressed an interest. This was a more formal process where we reviewed the research process and the main ethical concepts and consent forms. As a key ethical consideration in negotiating ongoing consent, the co-researchers and participants were made aware of their right to withdraw from the research at any point without obligation. None chose to withdraw.

### Confidentiality

The issue of confidentiality was, and remains, important in this research. The co-researchers, having dual roles as community members and researchers, may have come across information not generally accessible to the public during this study, specifically through the focus groups and interviews. This issue was addressed by discussing the principles of confidentiality with all co-researchers and participants and within the context of the research. Also, to ensure privacy, all co-researchers and participants were encouraged to choose a pseudonym, which was used in all cases referring to the research. It was also made clear to all the young people from the outset that there were limitations in being able to guarantee full confidentiality; for instance, if a safeguarding concern arose, the researchers would follow safeguarding procedures. Still, they were all informed that their responses would be confidential and that the results would be presented in such a way that no individual would be identified [45, 46].

### Reflective statement

As noted earlier, people live and communicate with others from various perspectives and positions that are shaped by intersecting aspects of social identities. In relation to this research, these aspects included: gender, age, education and religion, any or all of which may have impacted on young people’s experience during the research process. Therefore, prior to and during the research, the principal researcher and the co-researchers engaged in acts of reflexivity that critically examined their privilege, power and patterns of intentional and unintentional biases. Understanding and accurately representing intersecting positionalities in relation to community partners is essential for ensuring that researchers are authentically engaging in power-sharing, committed to co-learning and creating a positive collaborative experience [43, 47].

Furthermore, the goal of training young people to become facilitators in this study was to support them and the younger participants to express their views openly to their peer groups. This technique is promoted as a method that allows for a less hierarchical relationship between the researcher and the researched [47, 48], suggesting that young people would discuss topics amongst their peer more openly than they would with adult researchers [43]. This was indeed the case in the focus groups and interviews, where co-researchers and participants engaged in open discussions and, at times, co-researchers also participated in group exercises. This resulted in the breakdown of power, leading to deeper insights into young people’s lives.

### Data analysis

The data analysis after each phase of data collection followed the six-phase reflexive thematic analysis process described by Braun and Clarke [49, 50]. The three data corpora (training workshops, interviews and focus groups), were analysed using NVivo 11 software. Although conducting a robust analysis does not always require the use of customised software, its use enables transparency about how researchers go about their data analysis, by more easily illustrating the tasks engaged in, their sequence, role and documentation [51].

The data were analysed in the following way. First, the audio recordings were transcribed, followed by a process of reading and re-reading each transcript, assessing accuracy and identifying tentative themes and subthemes. This was followed by the process of identifying patterns across the data corpora (training workshops, interviews and focus groups). Initially, the principal researcher coded the data at a latent level, in which the analysis went beyond just describing the data, to identify the underlying ideas, assumptions and conceptualisations, thus aiming to gain a deeper understanding of it.

The authors then participated in a review of the initial codes identified from the data sets and discussed potential themes and subthemes emerging from the data. These were examined for strength and accuracy and to ascertain whether the interpretations, across the data, reflected what had been interpreted at the individual participant level. The fifth stage aimed to ensure there was clarity about each theme, and the results were categorised and subdivided into themes and subthemes containing units of data (quotations) to define them.

The final themes and subthemes were then reviewed by the authors as a form of member-checking to improve reliability and rigour and to ensure the findings were meaningful, accurate and met the research objectives. The original intention had been to involve the co-researchers in data analysis, and a specific event to do this was arranged. However, only one of the nine co-researchers was available, as the other eight co-researchers all had other commitments. Nonetheless, we arranged a follow-up meeting at a later stage to evaluate the training workshops. On that occasion, five co-researchers attended, and the results were shared and discussed with them.

### Establishing trustworthiness

This project followed specific measures related to credibility to demonstrate trustworthiness. To achieve credibility, this research utilised the following strategies: reflexivity, researcher debriefing (discussion of findings with co-researchers) and prolonged engagement (developing trust between researchers and research participants) [52]. Investigator triangulation and data triangulation were also used to ensure the trustworthiness of the findings, through the convergence of information from different sources [53, 54]. Denzin [40] notes that triangulation involves the employment of multiple external methods of data collection, as well as the analysis of those data. For this project, data were obtained from different sources (training workshops, interviews and focus groups), and the three different sites to achieve this (data triangulation).

## Results

A total of nine co-researchers and twenty participants were recruited. A summary of their backgrounds is shown in Tables 1 and 2.

**Table 1:** Background characteristics of nine co-researchers (aged 15–18)

Gender	Parent’s country of origin	Total
Female	Somalia	7
Female	Nigeria	1
Male	Somalia	1

**Table 2:** Background characteristics of twenty participants (aged 13–15)

Gender	Parent’s country of origin	Total
Female Male	Somalia	4 4
Female Male	Sudan	3 4
Female	Somaliland/Kuwait	1
Female	Egypt	1
Male	Yemen/Saudi Arabia	1
Male	Nigeria	2

**Fig. 1:**
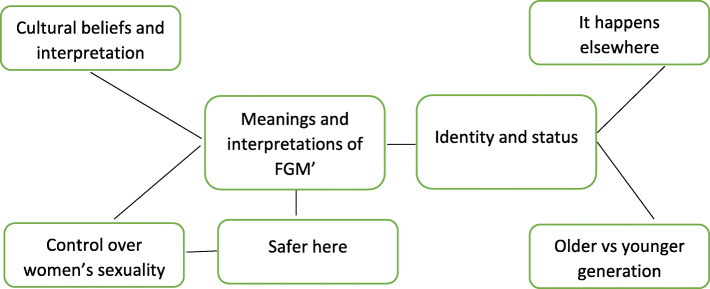
Overarching themes and subthemes of the research findings

Explorations of each theme and subtheme identified in the analysis are given below (see Fig. 1). The first theme, ‘meanings and interpretations of FGM’, forms the first half of the discussion, covering the subthemes: ‘cultural beliefs and interpretation’, ‘control over women’s sexuality’ and ‘safer here’. The second theme is ‘identity and status’, with the subthemes: ‘it happens elsewhere’ and ‘older vs younger generation’.

### Meanings and interpretations of FGM

This theme highlights participants’ responses when asked to define FGM, and illustrates how the level of exposure and understanding of this topic varied greatly among young people. Some responses were deeply personal, for instance, reflections on their awareness of family members who had undergone the procedure. Others defined FGM within the context of their own cultures, juxtaposed with their experiences of living in the UK, and how these experiences may have shaped or transformed their understanding it. These interpretations showed that, although participants acknowledged that FGM was embedded within their culture, they nevertheless examined the importance of their British social environment in discussions about whether FGM should be stopped, or whether it was possible to continue the practice, albeit in a context they perceived as ‘safer’. The most prominent finding is the presumption of safety – during the focus groups and interviews, participants discussed their understanding that more suitable and sterile equipment was used in the UK, which they believed made the procedure safer in this country.

#### Cultural beliefs and interpretation

The participants acknowledged the historic nature of FGM, stressing that raising awareness has changed attitudes towards it. Halimo discussed the important role that grandmothers play in shaping a family’s views on the tradition, and how this has changed through education and coming to the UK:

“I am not going to lie; I am Somali. I am African. So it is in my culture to do it [FGM] … it happened years ago, and it probably happened to my grandmother, but she is probably educated about it now and knows what it is. However, she wouldn’t want it to happen to younger people like me, because it is not really healthy. And I think she would want me to have a good childhood and that would probably just ruin it … Because you would just be traumatised” (Halimo, 14. Female).

In the extract below, Sabrin highlighted this shift in the custom, acknowledging that it used to be done for cultural reasons, but is now becoming ‘unacceptable and forbidden’:

“It [FGM] used to happen, I heard that, because it was done for cultural reasons, but now they’ve stopped it and are trying to stop it even more. Like obviously, back in my Nan’s day and Nan’s mum, it was a normal thing to get it done … it’s just part of the culture, but now it’s seen as *haram*, like unacceptable and forbidden” (Sabrin, 14. Female).

Although the quotes above illustrate changing attitudes towards FGM, Ikram discussed the celebrations that exist to mark the completion of the procedure, revealing the preservation of culture and highlighting the intergenerational hierarchy and power amongst women. Ikram reflected on her grandmother’s key role in instigating the cutting of her sister, which subsequently her mother went along with:

“I know in Sudan they dress up in traditional clothing and go to the doctors and have it [FGM] done, they then come home and have a party to show it had been done … I don’t know what it symbolises. My sister did it because my grandma wanted her to do it and my mum went on with it because my grandmother wanted it. I think I hadn’t been born yet or was too young to remember” (Ikram, 15. Female).

Felicia presented a distinct view of FGM. Though she agreed that it was a cultural practice, she linked it to self-esteem and societal expectations, suggesting that women would do it to look ‘clean and neater’:

“I think if someone has low self-esteem, she would probably tell people she had had it done to make herself feel better and for people to like her. I think it can happen anywhere, not just in Cardiff. It’s mostly done because of culture really. Some women do it to make themselves look good down there and like make themselves clean and neater down below …” (Felicia, 15. Female).

This theme shows that whilst participants acknowledged that living in the UK has played a role in changing their family members’ views of FGM, others considered how even the law may not be enough to stop it.

#### Control over women’s sexuality

Whilst the social norms that perpetuate FGM differ between affected communities; participants felt that FGM was generally carried out to secure control over the sexuality of girls and young women, specifically to prohibit them from engaging in pre-marital sexual intercourse. Even though most participants were against ‘cutting’, they appeared to support other, perceivably safer forms of controlling young women’s sexuality. As Ikram argued, there are other ways to stop girls from having intercourse:

“I get why it’s done, but I don’t want my children to go through that pain. I know purity is one of the reasons why it’s done, but I am sure there are other ways, like to stop girls from having sexual intercourse” (Ikram, 15. Female).

The requirement that young women abstain from sexual intercourse until marriage was also supported by Lucy, who felt that there should be other means of safeguarding young women’s virginity, for instance, another less invasive and intrusive and more effective method than performing FGM would be not allowing young girls to leave the house alone:

“Like, why would they do that? Instead of like doing this procedure, they could literally just find another way to stop that girl from having any [bad] behaviour towards boys. Like not letting her out of the house by herself, she can be accompanied by someone else” (Lucy, 13. Female).

It became apparent that the responsibility for not engaging in sexual intercourse is solely placed on girls, with no discussion about how young men are part of this decision-making process. Moreover, the retention of ideas about restricting girls’ mobility, for example, was deemed more acceptable – while some participants did not agree with FGM others such as Lucy, nevertheless assumed that young women’s sexual and social freedom should somehow be regulated.

Ibo interpreted FGM as an act of protection but also of control; he used the word ‘sew’ to signify his awareness of the act that represents Type III FGM. Again, Ibo failed to acknowledge the involvement of young men in engaging in sexual intercourse:

“They protect the woman’s genital parts because they may be from a certain tribe or something and they don’t want them to have intercourse, so they sew it up or whatever” (Ibo, 13. Male).

During these discussions of FGM as a form of control over women’s sexuality, Mo expressed an internal conflict, stating that, while parents may love their daughters, they might also be prepared to inflict such bodily harm. He questioned how parents, who were otherwise perceived as loving and caring, could essentially ‘harm’ their children:

“It made me wonder why people would do this to their child that they are meant to love and care for and not hurt and ruin their lives and everything … like, when the parents try to stop their children from doing bad stuff, but then it might harm them even more, and stop them from having families, kids and everything … Maybe like the father or parents may think, ‘Oh, you’re not allowed to have sex until you’re married’ and then, when you’re married you can take off the surgery, the thing, yeah” (Mo, 13. Male).

However, Mo used the term ‘surgery’ for FGM and suggested that a woman might be able to ‘take [it] off’ once married, demonstrating a lack of knowledge regarding the ability to reverse the procedure and the wider physical and psychological complications associated with it.

#### Safer here

This subtheme highlights notions of ‘safety’ derived from participants’ experiences and knowledge about FGM. Here we see how the private and social spheres intersect – some participants suggested that the provision of better medical equipment in the UK would make the procedure safer, while others suggested that a different form of FGM is being performed in the UK, one that does not involve infibulation.

Sophia discussed a possibly ‘acceptable’ version (in the UK) in comparison to being ‘sewn’, also known as infibulation, which, is practised ‘back home’. Although her knowledge of FGM was limited, Sophia associated FGM with torture if it was done against a person’s wishes:

“It happens in this country, but it’s different to the ones you get done back home … they use better equipment, and it’s like safer. I heard it’s different; I think there’s like two types and one of it you get sewed up, the ones here [UK] you don’t get sewn, not sure what they do … In my opinion, it’s like torture. It shouldn’t be done if the person doesn’t want it” (Sophia, 15. Female).

Sophia did not explicitly mention the perpetrators involved, using the term ‘they’ to describe who would use better equipment. Nasir was of the opinion that FGM is safer when performed by ‘best-trained doctors’ in the UK. He discussed this in contrast to developing countries, where he perceived that the procedure involved a risk that may lead to ‘life-changing problems’.

“I guess here is much safer because we are a high-income country, we have NHS and best-trained doctors, so I guess it’s much safer, but in low-income countries life expectancy is lower, so it can be a risk, and it can lead to, like, life-changing problems” (Nasir, 13. Male).

The hidden nature of FGM, coupled with its prohibition in the UK, may have deterred young people from coming forward and reporting the act, making it a challenging and complex issue that young people may not be willing to discuss, due to concerns about the legal implications for their parents. During this discussion about safety, Maria shared her experience of hearing about the practice and disclosed that her cousin had undergone FGM ‘here, two years ago’:

“I heard it last year, my family were talking about it, some of them agreed and like, and the others didn’t … Some of them were like, it is haram, and the others believed it was Sunna or something … my cousin also had it here, [UK] two years ago … she was 18 years old” (Sabrin, 13. Female).

Although historic, it was a significant finding that a family member had recently undergone FGM in the UK. Sabrin went on to explain the role of choice in decision making, where she claimed that sometimes a girl ‘decides to have it’, but within the context of pleasing her parents – this questions the extent to which young people have agency:

“I think for girls before she decides to have it, she should ask the doctor, and if the doctor said okay, then she would do it to please her parents …” (Sabrin, 13. Female).

The discussion of choice was continued by Lucy, who disapproved of FGM, but stated that, as long as it was not risky, then it was acceptable:

“When it comes to FGM itself, I don’t think its right. But if she feels like she must do it, as long as it’s safe and no problems will come from it, then she can, but it should not be risky” (Lucy, 13. Female).

Although FGM is illegal in the UK and is not performed within the NHS, Aaliyah articulated a process where the parents could ask a doctor to perform the procedure:

“She [Mum] said that here [UK] if a mother would really want her child to have it, she would take her to a doctor. I don’t think the doctors perform the procedure any more but yeah only if you really want your child to go through this, but I don’t think any mother would want their child to go through that” (Aaliyah, 13. Female).

The young people appeared to associate the safety of FGM with the use of better equipment and access to better-trained doctors in the UK. Moreover, Sophia added that the procedure conducted in the UK did not involve infibulation. Whilst concerned about the safety of the procedure itself; participants failed to mention the subsequent physical and psychological implications that are often associated with FGM – having articulated their understanding based on what they had heard or learnt from family members, who may not be aware of the associated complications.

### Identity and status

In the previous subtheme, participants acknowledged that FGM might be practiced in the UK, albeit in small numbers. The second main theme highlights the tension among participants’ responses, where some of them also ‘othered’ FGM, distancing themselves by place, claiming that it is a practice that only happens in Africa or to immigrants. The respondents also discussed the evolving nature of FGM – that, through a process of assimilation, the custom was becoming outdated in the UK, although they acknowledged its persistence in other countries. Here participants also spoke about the challenges they faced when attempting to engage with their parents, peers and siblings on this subject.

#### It happens elsewhere

The belief that FGM only happens in African countries or to immigrants may reveal a level of vulnerability for young people in the West, which may stem from a lack of knowledge. Adil highlighted the significance of how FGM is framed in Western public discourse, as something that only happens to immigrants and not British-born people:

“… ‘cause they think like … ‘We are English, they aren’t going to come here, and they won’t do it here’ … ‘cause I haven’t heard of a case that it’s happened to an English person, they think, ‘ah it’s these immigrants, it’s happening to them so why do we have to worry about it?’ They are really naïve about it, thinking they don’t want anything from them immigrants” (Adil, 15. Male).

In his interview, Mo explicitly stated that ‘it’ [FGM] ‘happened in Africa’, distancing himself by location:

“Well, my mum talks about it a lot, but apparently, it [FGM] happened in Africa” (Mo, 13. Male).

Ibo explained the importance of increasing youth awareness about FGM because he thought that if people were told that it could happen to any girl, they might be better able to protect themselves from it:

“Tell them it’s real and that it’s becoming a problem, that some may be affected, then they would start to listen and understand, that this is not just happening to people who are not like them, that it could happen to anyone” (Ibo, 13. Male).

Ibo explained the importance of framing FGM as a problem that ‘could happen to anyone’. This message may contribute to changing the narrative, from an issue that only affects women and girls who appear to be from a specific community to one that could affect any girl.

#### Older vs. younger generation

The extract below illustrates the diverse interpretations of FGM, where Mo, whose mother is a Somali refugee, interpreted it as a traditional practice but his friends, on the other hand, condemned it as child abuse. Mo’s comment illustrates the shift in how the second generation may perceive FGM:

“She [Mum] just said that it’s tradition. My friends told me it was illegal, and it is a type of child abuse; it could change people’s lives” (Mo, 13. Male).

Whereas Mo distinguished the change in beliefs, from ‘tradition’ to ‘child abuse’, Aziza explored this shift within the frame of the older versus the younger generation. She acknowledged how– given that she was born and raised in the UK – she was able to talk openly about it and to challenge such customs within her family:

“When I say women, I feel like it’s the old women. For a long time, my grandma glorified it, and now I feel like her views have changed, and I guess it’s because we live in the UK now and she’s learned like how it’s not right anymore. I think it’s because of like her grandchildren have been raised in the UK, and we don’t have the same opinions as they do in Somalia. Even like today with topics that are not to do with FGM or are controversial, like my sister or anyone else in the family would talk about it and say like no, this is not how it’s supposed to be” (Aziza, 15. Female).

In the same vein, Ikram described how young people were now ‘open’ to discussing ‘this stuff’ [FGM]:

“My generation and kids younger than me, we are more open to this stuff than how people are like your age [principle researcher], how they were when they were my age” (Halimo, 14. Female).

Maria noted that her ability to talk about FGM with her brother was due to being raised in a ‘modern country like the UK’:

“Like they would want to talk about it. I have a brother, and I know he is definitely not pro-FGM, but in general, I know not a lot of men would agree with it, especially if they are raised in a modern country like the UK” (Maria, 15. Female).

Although she did not explicitly mention having discussed FGM with her brother, Maria ‘definitely’ knew that he was against it. She went on to explain that, although her parents are aware that UK law prohibits FGM, many parents circumvent the law and children are taken abroad to ‘have it done’:

“But I think we should also hear it from the media because I feel like it still happens today and like I’ve heard my parents telling me about like children my age being sent off to Somalia, for example, to have it [FGM] done ‘cause obviously, it’s not legal here … I think it’s good that it’s not legal here. I’m not sure if it still is okay in Somalia, maybe it is, but I know it still happens” (Maria, 15, Female).

Maria then related a discussion she had had with her mum, where she learnt that force may, at times, be employed against girls who are unwilling to comply:

“In Somalia, it was like expected of girls to have FGM practised on them, even if like I don’t know, my mum would tell me how they would like, hold you down as they did it” (Maria, 15. Female).

Young people appeared to distance themselves from FGM by place, age group and origin – although they acknowledged that FGM is still happening in other countries, they discussed how living in the UK enabled them to feel liberated and to be able to talk about sensitive topics such as FGM.

## Discussion

The findings presented from this study suggest that young people’s interpretations of FGM are socially constructed and generated around norms which help them to define appropriate and inappropriate behaviours – because adhering to normative expectations helps to fulfil a person’s need to belong [55]. The layered levels of interacting systems in their development are shaped by norms and values embedded not only in the experiences of their family, peers and schools but also in broader social and cultural contexts [56]. In relation to this research, second-generation young people are influenced by two sets of norms – one from their cultural heritage, learnt from their parents, wider family and community networks, and the other from the mainstream culture, acquired through education, interactions with their peers and from the broader social context. These two sets of cultural values and ideals have the potential to be different and, at times, even conflicting. In their attempts to resolve this discrepancy, young people appear to have ‘othered’ FGM, by distancing it as being historical or a practice that is far removed from themselves, taking place in other parts of the world.

In recent years, the UK has seen some positive changes in terms of addressing FGM, not least because of collaborations between women’s organisations [57]. In particular, major cities such as London and Bristol have seen an increase in community groups campaigning against FGM [57]. Organisations such as Refugee Women of Bristol, Integrate UK, and the Foundation for Women’s Health Research and Development (FORWARD) have created spaces for women to voice their experiences of, and reject, FGM. However, it is also true that the UK government has recognised that targeted strategies are necessary to achieve a reduction in the number of girls at risk of being subjected to FGM. In 2015, the Serious Crime Act was introduced [58], which made it mandatory for regulated professionals to report any known cases of FGM. The problem here, and one which is specific to affected communities, is the failure to connect different areas of policy that address all women at risk, rather than women from FGM affected communities in isolation. Such an approach may inadvertently lead to stigmatising those communities. Dustin [59, 60] echoes similar concerns in relation to policies aimed at preventing Violence against Women and Girls (VAWG).

This unidimensional account may have resulted in participants formulating the concept of ‘otherness’, by differentiating themselves from such victims, using terms such as ‘we/they’ and ‘us/them’ and changing the narrative from FGM being something ‘we’ do, to something ‘other’ people do, people different from them by place, age and custom. As such, participants in this research critiqued the anti-FGM messages they had been exposed to, claiming that FGM should be framed as an issue that could affect anyone, moving away from reductive messages that suggest FGM only impacts certain communities. Montoya and Agustin [61] have argued for a shift towards framing immigrant cultural practices within the wider framework of violence against women and girls, asserting that redefining FGM as violence against women and girls rather than a cultural tradition would assist in preventing the stigmatisation of communities as barbaric others.

Against this backdrop, we argue that, amongst second-generation young people in the UK, understandings of FGM are entangled within a web of complex attitudes and beliefs influenced at multiple levels by diverse sources, ranging from personal knowledge, attitudes, emotions and risk perceptions, to social issues, including body image. Therefore, presenting the issue solely within the violence against women framework is unhelpfully reductive, because it fails to take account of the complexities young people face. Context thus becomes a crucial part of the discourse surrounding FGM in the West. This study shows that, where specific forms of violence such as FGM are framed as traditions enacted on specific groups of women in particular parts of the world, young people may inadvertently counter this narrative by perceiving forms of FGM that are evident in the West to be safer, therefore reducing their perception of its danger.

Although there have been several interventions in the UK intended to prevent FGM – health, policy and community-led – we argue that these initiatives may have limited impact if they do not understand the context in which the custom endures. This suggests that future strategies to tackle and prevent FGM and to bring about cultural change need to embrace a holistic, intersectional approach, one that is relevant to young people in the UK and is guided by their values, beliefs, perceptions and everyday lives.

A core principle for such an intersectional approach is acknowledging that knowledge development must come from the perspective of the oppressed, not the dominant group [62]. Such an upstream approach responds to the social constructions of race, class and gender being unequal in social relationships, and would be more effective than any approach limited to prevention and lifestyle, which excludes the importance of the social dimension. Therefore, we should strive for negotiated [63] and community-focused, rather than authoritative initiatives, to empower people to make healthier choices. This requires the use of activities focused on the ability to strengthen collective participation and action, which is perhaps identical to the broad tradition of community development and, in particular, the ‘bottom-up/collective’ approach termed community action [64]. This would consolidate, challenge and reverse the core motivations for carrying out FGM from a second-generation perspective, and thereby contribute towards eradicating this harmful act, by removing it from being one of the key cultural factors of specific communities.

### Strengths and limitations

The main strength of this study was the success at involving young people as co-researchers, which enabled us to gain an in-depth insight into young people’s attitudes and perceptions of FGM. The co-researchers described the research process as a positive experience and reported that they had gained new skills, which many of them have adopted at school and work. Also, the non-judgmental and supportive space created during the project not only enabled young people to share and exchange their knowledge of FGM but also potentially to challenge FGM in their home lives. However, it is essential to recognise the complexity of collaborating with young people and accept that it is often difficult to commit fully to a collaborative process. Young people experienced time constraints and limited control over their own schedules and availability. Parental expectations and rules were also outside their control. These restrictions, coupled with the demand of academic timeframes, limited their participation during data analysis, although they were able to discuss the findings during the evaluation stage.

## Conclusion

This study indicates that several factors influence second-generation young people’s attitudes towards, and perceptions of, FGM. These range from social norms and cultural values obtained from their immediate family network to their wider network in the UK. Although the young people held negative perceptions of FGM, current narratives in the West may have contributed to the shift in how they interpreted its safety, that is, the participants felt that the West offers safer options for undergoing FGM due to having better equipment, perhaps not directly linked to the medicalisation of the practice, thereby situating FGM as a less harmful procedure if carried out in the UK. This research offers a starting point, by providing narratives from the second generation, indicating a shift beyond harm reduction approaches towards those that engage the respective communities; by creating positive and inclusive ways to work ‘with’ communities on this issue rather than ‘on’ them, in line with co-productive processes.

## Supplementary information

**Additional file 1.** Focus group guide

**Additional file 2.** Interview guide

## Data Availability

Due to privacy restrictions, the data sets generated and analysed in the present study are not publicly available, as they contain information that may compromise the participants’ anonymity.

## Abbreviations

CBPR: Community-based participatory research
BAME: Black, Asian and Minority Ethnic
FGM/C: Female genital mutilation/Cutting
RSE: Relationship and Sex Education
WHO: World Health Organisation
UNICEF: United Nations Children’s Fund
