# Supplementary material for: Exploring young people’s interpretations of female genital mutilation in the UK using a community-based participatory research approach
Source: BMC Public Health. 2020 Jul 20;20:1132. doi: 10.1186/s12889-020-09183-6 (PMC7370427; doi:10.1186/s12889-020-09183-6)
Supplement: Supplementary file 1 — Additional file 1. Focus group guide [file 12889_2020_9183_MOESM1_ESM.docx]

**Additional File A: Focus group guide.**

**Allow 2 hours**

1. **First-round-Approximately 30 minutes**

Introductions followed with an ice breaker

Now we have introduced each other, can you say briefly, why you volunteered to join this focus group?

**Healthy-self drawing game**

Now, everyone has a paper each; we would like what you visualise your version of a happy young man or woman and draw them.

Probe: Consider who is around them, where they live, dress code, body language. What makes them healthy and happy?

1. **Second round- Approximately 1hr**

I have placed post-it notes on the table; I would like you to write if you have heard of the term FGM, FGC or circumcision. What did you hear? Do not include your name, place the completed cards on the floor. As a group, use the notes to debate, true or false.

1. We would like you to draw a young person who has never heard of FGM, what questions would they have and how would it make them feel?

Probe: write down their thoughts, feelings and questions they might have.

1. Now, what do other people say about FGM? If they were to explain it to their peers, what terms would they use? Write them down and then place them on the floor upside down. Then, as a group, discuss what FGM is.
2. Lastly, draw a person who has heard of FGM, how do they feel, what questions would they have, what do they know about it?
3. **Approaches**
4. In pairs, write down where young people learn about FGM- place on the floor — Venn Diagrams (the bigger the circle, the more significant).

**Use these to discuss, content, age and their views.**

1. Have you had contact with any campaigning groups?

Which ones were they? What are your views about them?

**Legal mechanisms:**

The FGM Act 2003, the Serious Crime Act 2015

Where do you think the law stands in terms of FGM in the UK? Why do you think it is?

**Health Risks**: Give a list of random health complications, ask participants to mark which ones they think should relate to FGM. Then discuss these as a group.

**Positive deviance:**

Have any of your friends discussed FGM with you?

What was the discussion? How old were you when you heard about FGM?
