# Supplementary material for: Exploring young people’s interpretations of female genital mutilation in the UK using a community-based participatory research approach
Source: BMC Public Health. 2020 Jul 20;20:1132. doi: 10.1186/s12889-020-09183-6 (PMC7370427; doi:10.1186/s12889-020-09183-6)
Supplement: Supplementary file 2 — Additional file 2. Interview guide [file 12889_2020_9183_MOESM2_ESM.docx]

**Additional file B: Interview guide**

**General questions**

My name is (x), I will be conducting the interview today. Let us start with your name?

Tell me about yourself?

Probe: What school do you go to? What do you enjoy doing?

- What about home, how many brothers and sisters do you have?
- What about friends? Any that you have at school and home?
- What do you think are the expectations of you growing up?
- What things do you worry about growing up?

1. **Section A: Beliefs and perceptions around FGM**

- Have you heard of the term FGM OR Female circumcision? What have you heard?
- Do you remember how old you were when you learnt about the term? Probe: Where did you hear of it?
- In your opinion, what is your understanding of the term? What does it mean to you?

1. **Section B: Attitudes**

- So, when you first heard about it, how did you feel?
- Do you think attitudes to FGM differ between females and males? If so, how?

1. **Section C: Relationships (Family dynamics)**

- Have you spoken about FGM with anyone in your family?

If so, how did you find the conversation went? Whom did you speak to?

- From your background, i.e. where your parents are from, have you heard any rules or expectations on FGM?
- Whom would you talk to about the subject? Why?

1. **Section D: Barriers and challenges**

- What, in your opinion, would be the main barrier in talking about FGM with people your age? In other words, what would stop you from talking about it?
- How do you think we can overcome this? (How can it be made easy for young people like you to talk about it)?
- How do you think we could help young people know or understand more about FGM?

Probe: Imagine someone who has never heard about this subject, how can we make them understand it? What questions do you think they would have?

Probe: Any suggestions on how to approach young people, teach them?

- How would you have liked to learn about it?
- Would you talk about FGM with your friends? If so, why? If not, why?

**Section E: Any other issues**

I think that is everything I had to ask you to talk about, have you got anything else you would like to say or any final thoughts or anything you would like to follow up that I have not asked you

**End of Interview- Thank interviewee for their time**
